# Presence of Anxiety and Depression in Patients with Open-Angle Glaucoma of Different Degrees of Damage

**DOI:** 10.3390/jcm14113954

**Published:** 2025-06-03

**Authors:** Marija Olujić, Dubravka Biuk, Slaven Balog, Ivana Kotromanović Šimić, Darko Kotromanović, Katarina Dodig-Ćurković

**Affiliations:** 1 Ophthalmology Polyclinic Dr. Balog, Ivana Gundulića 36 b, 31000 Osijek, Croatia; molujic9@gmail.com (M.O.); slavenbalog@gmail.com (S.B.); 2 Faculty of Medicine Osijek, Josip Juraj Strossmayer University of Osijek, Josipa Huttlera 4, 31000 Osijek, Croatia; ivana.simic.osijek@gmail.com (I.K.Š.); kotromanovic93@gmail.com (D.K.); katarina5dodig@gmail.com (K.D.-Ć.); 3 Clinic for Eye Diseases, Clinical Hospital Centre Osijek, Europska Avenija 14, 31000 Osijek, Croatia; 4 Oncology Clinic, Clinical Hospital Centre Osijek, Josipa Huttlera 4, 31000 Osijek, Croatia; 5 Institute for Child and Adolescent Psychiatry, Clinical Hospital Centre Osijek, Europska Avenija 14, 31000 Osijek, Croatia; 6 Faculty of Dental Medicine and Health Osijek, Josip Juraj Strossmayer University of Osijek, Crkvena 21, 31000 Osijek, Croatia

**Keywords:** anxiety, depression, glaucoma, open-angle, intraocular pressure, mental disorders, mental health, quality of life, stress, psychological, surveys and questionnaires

## Abstract

**Background:** Glaucoma is a group of eye conditions that damage the optic nerve, and it can be interconnected with psychoneurotic disorders due to the psychological and emotional stress that comes with such a chronic condition. The aim of this study was to examine the characteristics of the occurrence of anxiety, depression and open-angle glaucoma (OAG) in glaucoma patients from December 2023 to December 2024. **Methods:** This cross-sectional study was conducted on 200 patients with three different stages of OAG. Multiple questionnaires were used to determine the influence of different OAG stages on the severity of anxiety and depression. **Results**: While predicting the anxiety expression, in a multivariate logistic regression (stepwise method), there was a significant model in predicting the expression of anxiety: female gender (Odds ratio (OR) = 3.03), age of 66 and over (OR = 3.4) and the feeling of being under stress (OR = 7.07). In the prediction of a higher severity of depression, predictors are age 66 and older (OR = 2.03) and feeling stressed (OR = 9.47). **Conclusions**: While glaucoma and psychoneurotic disorders affect different systems in the body, the psychological toll of living with glaucoma can lead to exacerbation of glaucoma.

## 1. Introduction

Glaucoma is the second most common cause of blindness, but it is the leading cause of irreversible blindness worldwide. It is a multifactorial chronic and progressive optic neuropathy [1,2,3] with common characteristics that result in progressive and irreversible deterioration of the optic nerve and retinal nerve fibres, with corresponding deficits in the visual field (VF) [1,2,3,4].

Since glaucoma is a progressive chronic disease, it is essential to recognize the disease as early as possible so that the patient can be treated before irreversible structural changes occur [5]. Accordingly, early diagnosis is a priority. Antiglaucoma (ATG) treatment aims to slow down and/or prevent further progression of the disease. For now, ATG treatment with available medications and/or surgical therapy cannot reverse the prior glaucomatous damage to the optic nerve [3]. Glaucoma is most often a chronic disease that requires lifelong treatment and monitoring of possible disease progression [3]. Therefore, it is a significant socioeconomic problem, but also a psychological burden for the patient, since all forms of glaucoma can end in a complete loss of vision, i.e., blindness [2,6]. In patients with glaucoma, it is observed that even a slight loss of the peripheral part of the VF and the associated expected reduction in contrast sensitivity can have an impact on the patient’s functioning in everyday life, habits and quality of life, i.e., reduced opportunities for physical activity, impaired daily work habits and increased risk of injuries and falls [7].

Elevated intraocular pressure (IOP) is currently the only modifiable risk factor for controlling the progression of glaucoma [2]. Normal IOP is considered to be between 10 mm of mercury (mmHg) and 21 mmHg, with a mean of 15.7 mmHg [8] measured by Goldmann applanation tonometry (GAT), the gold standard for measuring IOP [9]. Only 2% of the world’s population has been shown to have normal values above 21 mmHg [10]. Traditional ATG treatment is based on lowering IOP with various ATG medications [8]. This is done to prevent the development of glaucomatous optic neuropathy. Although elevated IOP levels play a significant role in glaucoma, IOP alone is not always the determining factor.

Depending on the way in which the blockage of the aqueous humor drainage occurs, glaucoma is divided into open-angle glaucoma (OAG)—characterized by an open iridocorneal angle [11] and angle-closure glaucoma (ACG)—an anatomical configuration of the iridocorneal angle in which there is mechanical blockage of the trabecular meshwork by the peripheral part of the iris [12,13,14,15,16,17]. An additional division is into primary and secondary OAG and ACG [12,13,14,15,16].

Primary open-angle glaucoma (POAG) is a chronic, progressive disease of still unclear etiology, which can result in irreversible blindness [18]. It is usually encountered in patients older than 40 years of age and the likelihood of the disease increases with age. The following are at higher risk for developing the disease: older age, higher IOP values, people of black race, a positive family history of glaucoma in the first generation, people who are moderately to highly myopic (moderate to high myopia), low blood pressure—diastolic, thinner central corneal thickness [13,18].

OAG can be divided into three stages: early, moderate and advanced glaucomatous loss. In early glaucomatous loss, the target IOP range is between 15 mmHg and 17 mmHg, in intermediate glaucomatous loss the target IOP range is between 12 mmHg and 15 mmHg and in advanced glaucomatous loss the target IOP range is between 10 mmHg and 12 mmHg [18,19]. It is of utmost importance to maintain normal IOP values in patients with glaucoma. Patients with glaucoma are truly concerned about the health of their eyes—they fear blindness and increased financial costs due to long-term use of ATG therapy, but also a decrease in the ability to perform daily activities, including work. It has been shown so far that chronic diseases, such as glaucoma, are associated with psychological disorders, most often depression and anxiety, and therefore many studies have been conducted that have shown that the prevalence of anxiety or depression is high in patients with glaucoma [20].

Although studies have shown that the disease contributes to the development of anxiety and/or depression, few studies have so far indicated that negative emotions, such as anxiety or depression, are also a risk factor for the progression of physical diseases [21]. Anxiety and depression, as psychiatric disorders, account for an estimated 13% of the global burden of disease and are among the most difficult diseases to treat. These disorders typically present with a diverse range of symptoms, have complex genetic risk associations, and poorly understood aetiology [22]. Along with this, it has also been noted that patients with glaucoma often share common character traits, such as an irritable and excitable temperament associated with perfectionist efforts and signs of neuroticism, but also segments of the tendency to develop anxiety, irritability and hypochondria [21,23,24,25,26,27]. According to some studies conducted so far, these character traits have proven useful in the treatment of glaucoma because patients adhered to the correct application of ATG therapy in the treatment of glaucoma, while, on the other hand, these character traits have proven to be a risk factor for the development of glaucoma [24,28,29]. Poor adherence to prescribed ATG therapy was observed in patients with developed anxiety symptoms, due to previously known low bias towards ATG therapy [20].

The aim of this study was to examine the characteristics of the occurrence of anxiety, depression and open-angle glaucoma in glaucoma patients.

## 2. Materials and Methods

### 2.1. Participants (Respondents)

This cross-sectional study included 200 patients diagnosed with three different stages of POAG who were treated at the Glaucoma Infirmary of the Clinical Hospital Centre Osijek in a one-year period from December 2023 to December 2024. Patients who were included were those who came for a check-up due to POAG and had treated for more than two years, as well as those who were diagnosed with POAG within the past two years. Inclusion criteria were patients with POAG with ATG therapy, adults aged 18 to 70 years, while the exclusion criteria were underage people or those over 70 years of age, those with a history of glaucoma surgery or a history of iridotomy, as well as people not being treated for POAG.

All patients included in this study underwent a complete ophthalmological examination, which consisted of taking a detailed medical history, examining the anterior and posterior segment of the eye with a slit-lamp biomicroscope, measuring IOP with a GAT, and additionally completing the questionnaires.

POAG staging was based both on the severity of VF damage and structural metrics of the optic nerve head. A staging system was thus based on the mean deviation (MD) parameter of the VF, i.e., early glaucomatous loss MD ≤ 6 decibel (dB), moderate glaucomatous loss 6 > MD ≤ 12 dB and advanced glaucomatous loss MD > 12 dB [18].

### 2.2. Questionnaires

The research was conducted with the Crown–Crisp Experiential Index (CCEI), which is intended to identify and measure common symptoms and personality traits within the conventional categories of psychoneurotic diseases and personality disorders. It is used to determine psychoneurotic disorders, and consists of 48 questions, which include six subscales: free-floating anxiety (FFA), phobic anxiety (PHOA), obsessiveness, somatic manifestations of anxiety, depression and hysteria. The total score provides a measure of general emotional instability or neuroticism with a profile of six subscale scores [30,31,32]. The CCEI questionnaire was translated into Croatian, and published by “Naklada Slap”, which gave written permission for the use of 200 copies of the CCEI questionnaire for the purpose of this research. CCEI has undergone all appropriate linguistic and cultural validation processes to ensure its reliability and validity in the Croatian population.

The General Anxiety Disorder 7 Scale (GAD-7) is a validated instrument for identifying generalized anxiety disorder [33]. It consists of seven questions that are answered on a Likert scale from 0 (“not at all”) to 3 (“almost every day”), depending on how often one of the listed disorders has occurred during the past two weeks. The total score can range from 0 to 21 and indicates the severity of the anxiety disorder. The results can be divided into four categories of anxiety: mild (0–4), moderate (5–9), moderately severe (10–14) and severe anxiety (15–21) [33,34]. The third questionnaire was the Patient Health Questionnaire 9 (PHQ-9), which is used for detecting depressive disorders [35]. It consists of nine items that address symptoms of depression (e.g., decreased interest in doing usual activities, feeling down, depressed, or hopeless, trouble sleeping, feeling tired or lacking energy, problems with appetite, feeling dissatisfied with oneself, difficulty concentrating, being slow or moving excessively, and thoughts of death and self-harm). Respondents indicated the frequency of occurrence of the listed symptoms during the past two weeks on a Likert scale from 0 (“not at all”) to 3 (“almost every day”). The total score of the PHQ-9 questionnaire ranges from 0 to 27. Based on the total score, the results can be classified into five categories depending on the degree of depression: minimal (0–4), mild (5–9), moderate (10–14), moderately severe (15–19) and severe depression (20–27). The GAD-7 and PHQ-9 questionnaires have been translated into Croatian, are publicly available, and their use does not require permission [33]. A schematic comparison of CCEI, GAD-7 and PHQ-9 questionnaires by function, usability and clinical validation is shown in Table 1. Before administering the CCEI, GAD-7 and PHQ-9 questionnaires, the respondents were familiar with their content and purpose, and they signed an informed consent and consent for their administration and data collection. In addition to using the questionnaire, the following information was examined and recorded on the questionnaire: gender of the participants, age of the participants, POAG stage, length of treatment for POAG, number of application of ATG therapy per day, whether the patient achieved the target IOP with ATG treatment, whether they suffered from other ophthalmological conditions before the diagnosis of POAG and whether they felt stressed.

This study has observed the associations between the observed variables without interfering the treatment; accordingly, the patients did not receive mental health referral whether or not significant anxiety and/or depression scores were detected.

### 2.3. Statistical Methods

Before we have started this research, we used G*Power (ver. 3.1.2.) to determine the minimum required sample size for three independent groups, with a significance level of 0.05 and a power of 0.80. The minimum required sample size was 159 subjects. For regression analysis, the minimum required sample size with a power of 0.85 is 129 subjects. In conclusion, the minimum causal size is 159 subjects (58 per group).

Categorical data are presented as absolute and relative frequencies. Differences in categorical variables were tested with the χ^2^ test and, if necessary, with Fisher’s exact test. Normality of distribution of continuous variables was tested with the Shapiro–Wilk test. Continuous data were described by the median and interquartile range (IQR) limits. Differences in continuous variables between two independent groups were tested with the Mann–Whitney U test (the Hodges Lehmann difference in median with the corresponding 95% confidence interval for the difference is expressed). Differences in continuous variables between three independent groups were tested with the Kruskal Wallis test (with the Conover post hoc test). The correlation score is given by the Spearman correlation coefficient ρ (Rho). Logistic regression, bivariate and multivariate (Stepwise method), evaluated the influence of several factors (stages of glaucoma and other general and clinical characteristics) on the probability of given outcomes: expression of anxiety and depression [38,39]. All values are two-sided. The significance level was set at alpha = 0.05. The statistical package MedCalc^®^ Statistical Software version 23.1.1 was used for statistical analysis [40]. The report on the conducted research was prepared according to the guidelines for reporting research results in biomedicine and healthcare [41].

### 2.4. Study Approval

The study was approved by the Ethics Committee of the Clinical Hospital Centre Osijek (approval date: 11 November 2022, Number: R1-14554/2022) and the Ethics Committee of the Faculty of Medicine Osijek (approval date: 23 February 2023, Class: 602-04/23-08/03, Reg. Number: 2158-61-46-23-17). Informed consent for voluntary participation in the study was obtained from all eligible patients who met the appropriate inclusion criteria.

## 3. Results

### 3.1. Basic Characteristics of the Participants

This study was conducted on 200 subjects, patients treated for POAG, of whom 68 (34%) were men and 132 (66%) were women. Most subjects were aged 56 to 65. The response rate of the participants was 100%. Previous ophthalmological diseases were recorded in 36 (18%) subjects. The number of subjects was equal for all three stages of POAG. A total of 93 (46.5%) subjects had a subjective feeling of stress. A total of 150 (75%) subjects achieved the target values of IOP (Table 2).

The median age of the subjects was 65 years, ranging from 37 to a maximum of 70 years, and the median duration of treatment was 96 months (8 years), ranging from one month to 564 months (47 years). Subjects received ATG therapy one to four times per day, with a median of two times (Table 2).

The median total score of the GAD-7 scale is 8, ranging from 0 to 20. According to the achieved values, it is observed that 55 (27.5%) respondents have a mild level of anxiety, and moderately severe or severe anxiety is recorded in 81 (40.5%) respondents (Table 2).

The median total score of the PHQ-9 scale is 7, ranging from 0 to 22. According to the achieved values, it can be seen that 69 (34.5%) respondents have a minimal level of depression, 38 (19%) have a moderate level and moderately severe or severe depression is recorded in 31 (15.5%) respondents (Table 2).

### 3.2. Correlations Between Glaucoma Stages and Anxiety (GAD-7) Correlation of Respondents’ Anxiety (GAD-7 Questionnaire) with Respondents’ Characteristics

The anxiety score is significantly higher in respondents who report feeling stressed than in those who do not feel this way (Mann–Whitney U test, *p* < 0.001), while there is no significant differences in the anxiety score according to the GAD-7, which assesses other characteristics of the respondents (Table 3).

By examining the correlation, it was observed that the age of the subjects is significantly, positively and weakly correlated with the GAD-7 questionnaire score in the group of subjects who achieved target IOP values (Rho = 0.215), while there is no significant correlation between the GAD-7 score and the length of treatment and the degree of glaucoma (Table 4).

**Figure 1:**
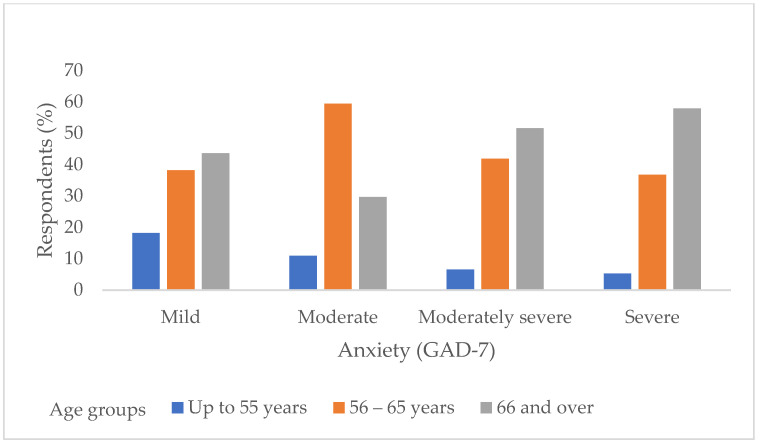
Distribution of respondents according to age groups in relation to the severity of anxiety determined by the GAD-7.

**Figure 2:**
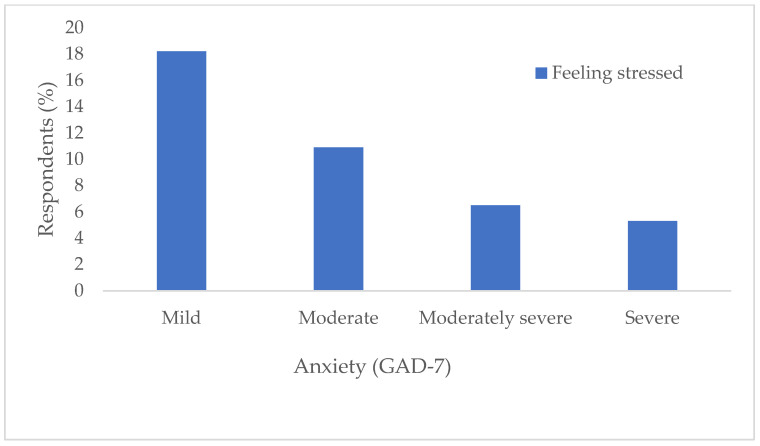
Distribution of respondents according to the feeling of stress in relation to the severity of anxiety determined by the GAD-7.

In the correlation of anxiety levels according to the GAD-7 questionnaire, it is observed that moderately severe and severe anxiety is significantly more pronounced in subjects aged 66 and over compared to younger subjects (χ^2^ test, *p* = 0.04) (Figure 1). Also, out of a total of 93 (47%) subjects who feel stressed, there are significantly more subjects, 16 of them (84.2%) with moderate or severe depression (χ^2^ test, *p* < 0.001) (Figure 2).

### 3.3. Correlations Between Glaucoma Stages and Depression (PHQ-9) Association of Depression (PHQ-9 Questionnaire) with Characteristics of the Subjects

**Figure 3:**
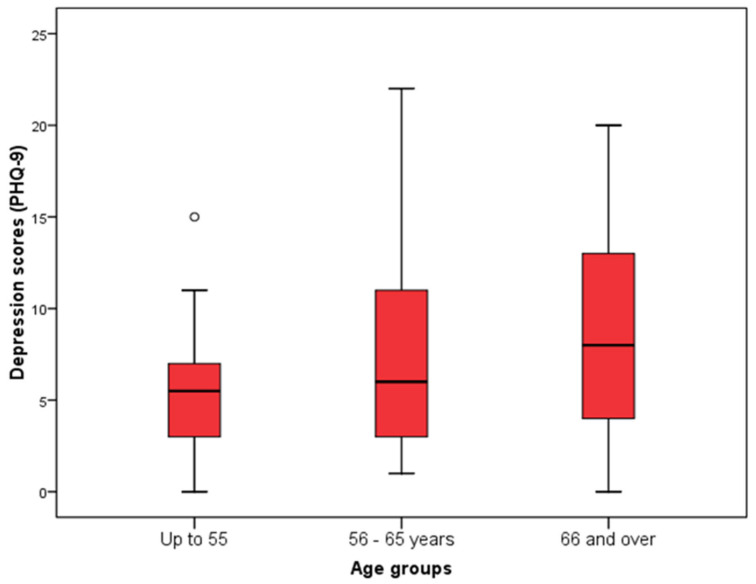
Distribution of respondents according to age groups in relation to the depression scores determined by the PHQ-9.

**Figure 4:**
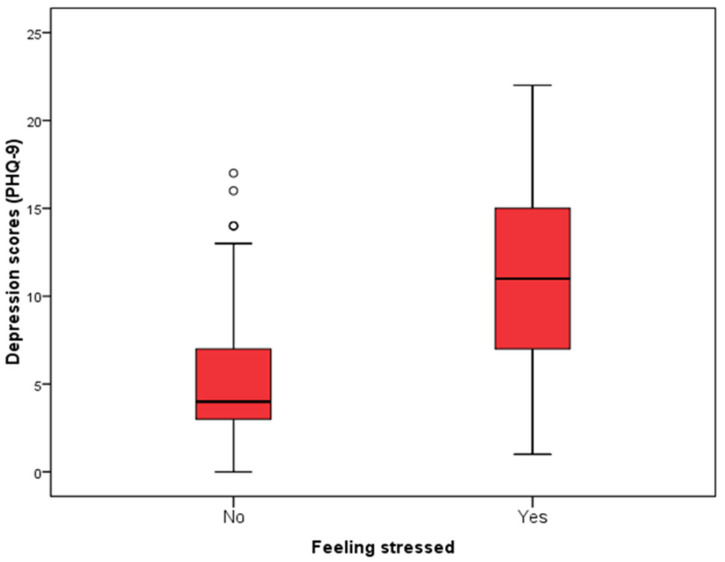
Distribution of respondents according to the feeling of stress in relation to the depression scores determined by the PHQ-9.

The lowest depression score (according to PHQ-9) was significantly observed in subjects aged up to 55 years compared to subjects aged 66 and over (Kruskal Wallis test, *p* = 0.03) (Figure 3). Also, the depression score was significantly higher in subjects who reported feeling stressed than in those who did not (Mann–Whitney U test, *p* < 0.001), while there were no significant differences in depression scores according to other characteristics of the subjects (Figure 4, Table 5).

Spearman’s correlation coefficient was used to assess the relationship between the depression score according to the PHQ-9 questionnaire and the age of the subjects and the duration of the disease (months). It was noticed that the age of the subjects is significantly, positively and weakly related to the result of the PHQ-9 questionnaire, in the entire group of subjects (Rho = 0.182), as well as in the group of subjects in whom the target IOP values were achieved (Rho = 0.269).

The same significant relationships are also found in the case of the length of treatment. The longer the treatment, the more pronounced the depression in the group of all subjects (Rho = 0.199), and in the group of subjects in whom the target IOP values were achieved (Rho = 0.221). The degree of glaucoma is not significantly related to the depression score (PHQ-9 scale) (Table 6).

In the correlation of the level of depression according to the PHQ-9 questionnaire, it is observed that out of a total of 93 (47%) respondents who feel stressed, there are significantly more respondents, 29 of them (93.5%) with moderate or severe depression (χ^2^ test, *p* < 0.001).

### 3.4. Correlations Between Glaucoma Stages and Psychoneurotic Disorders (CCEI)

With regard to the results of the CCEI questionnaire, it can be seen that the highest score was given to obsessiveness and somatic manifestations of anxiety and depression, and the lowest to the hysteria subscale (Table 7). 

Considering the obtained values, 98 (49%) of the respondents had pronounced depression, 81 (40.5%) had pronounced FFA, while the least of them, 42 (21%) had pronounced obsessiveness (Table 8).

### 3.5. Association of Psychoneurotic Disorders (CCEI Questionnaire) with the Age of the Subjects, the Duration of Treatment and the Stage of Glaucoma

Spearman’s correlation coefficient was used to assess the correlation between the age of the subjects, the duration of treatment and the stage of glaucoma with the subscales of the CCEI questionnaire. It is observed that the significant correlations are somewhat weaker (Rho < 0.5).

In the group of all subjects, older subjects have a more pronounced somatic manifestation of anxiety (Rho = 0.190) and depression (Rho = 0.183). The longer the treatment, the more pronounced the somatic manifestation of anxiety (Rho = 0.164), and the lower the stage of glaucoma, the more pronounced obsessiveness (Rho = −0.147).

In subjects who did not achieve the target IOP, older subjects have a less pronounced FFA (Rho = −0.344), and subjects with a higher stage of glaucoma have a more pronounced hysteria subscale (Rho = −0.323).

Respondents with achieved target IOP and older respondents have lower values of FFA (Rho = 0.174), less pronounced somatic manifestations of anxiety (Rho = 0.222) and less pronounced depression (Rho = 0.251). The longer the duration of treatment, the more pronounced the somatic manifestation of anxiety (Rho = 0.197) (Table 9).

### 3.6. The Influence of Glaucoma Stage and Other Characteristics of the Subjects on the Severity of Anxiety and Depression

To check the influence of glaucoma stages and other characteristics on the severity of anxiety and depression, bivariate and multivariate logistic regression were performed.

In the prediction of a higher severity of depression according to the PHQ-9, in the bivariate logistic regression, it is observed that older patients (odds ratio (OR) = 1.08) and those who feel stressed (OR = 9.61) increase the probability of a more pronounced depression (PHQ-9). Multivariate logistic regression (stepwise method) shows that there is a significant model in the prediction of the severity of depression according to the PHQ-9, and it consists of two predictors: age 66 and older (OR = 2.03) and feeling stressed (OR = 9.47). The model is fully significant (χ^2^ test = 53.1, *p* < 0.001) and explains from 23% (according to Cox&Snell R^2^) to 32% (according to Negelkerke R^2^) of the variance in the severity of depression and correctly classifies 74% of cases (Table 10).

In predicting higher levels of anxiety according to GAD-7, in bivariate logistic regression, it is observed that the feeling of being exposed to stress (OR = 6.02) and age in the range of 56 to 65 years (OR = 2.82) increase the probability of higher levels of anxiety (GAD-7). Multivariate logistic regression (stepwise method) shows that there is a significant model in predicting the level of depression according to GAD-7, and one predictor is the feeling of being under stress (OR = 6.02). The model is completely significant (χ^2^ test = 26.2, *p* < 0.001) and explains from 12% (by Cox&Snell R^2^) to 18% (by Negelkerke R^2^) of the variance in the level of anxiety and correctly classifies 73% of cases (Table 10).

In the prediction of the expression of FFA according to CCEI, in bivariate logistic regression, it is observed that women compared to men (OR = 1.98), subjects of older age (OR = 1.05) and those subjects who feel that they are under stress (OR = 6.05) increase the probability of more pronounced FFA. Multivariate logistic regression (stepwise method) shows that there is a significant model in predicting the expression of FFA, and it consists of the following predictors: female gender (OR = 3.03), age of 66 years and over (OR = 3.4) and the feeling of being under stress (OR = 7.07). The model is entirely significant (χ^2^ test = 43.5, *p* < 0.001) and explains from 22% (according to Cox&Snell R^2^) to 30% (according to Negelkerke R^2^) of the variance in expression of FFA and correctly classifies 70% of cases (Table 10). 

In the prediction of the severity of PHOA according to CCEI, in bivariate logistic regression, it is observed that women compared to men (OR = 3.31), older respondents (OR = 1.07), and those respondents who feel stressed (OR = 2.45) increase the probability of more pronounced PHOA. Multivariate logistic regression (stepwise method) shows that there is a significant model in the prediction of the severity of PHOA, and it consists of three predictors: female gender (OR = 6.12), older age (OR = 1.08) and feeling stressed (OR = 3.33). The model is entirely significant (χ^2^ test = 35.9, *p* < 0.001) and explains from 19% (according to Cox&Snell R^2^) to 26% (according to Negelkerke R^2^) of the variance in the severity of PHOA and correctly classifies 71% of cases (Table 10).

### 3.7. Correlation of the Depression Subscale with the Characteristics of the Subjects

In the depression subscale, there is a significantly higher score in women than in men (Mann–Whitney U test, *p* = 0.006), and in subjects who state that they feel stressed than in those who do not (Mann–Whitney U test, *p* < 0.001). Respondents under the age of 55 have a significantly lower score than older respondents (Kruskal Wallis test, *p* = 0.007), while there are no significant differences in the scores of the somatic manifestation of anxiety subscale in terms of other characteristics of the respondents (Table 11).

Significantly more respondents aged 66 and over (χ^2^ test, *p* = 0.001) and those who feel stressed (χ^2^ test, *p* < 0.001) have pronounced depression, while in other characteristics there are no significant differences in the distribution of respondents according to the severity of depression (Table 12).

## 4. Discussion

Glaucoma is a group of eye conditions that damage the optic nerve, which leads to optic neuropathy [4]. It is often associated with IOP, although it can also occur with normal IOP [2]. There is some evidence that chronic stress can have a negative effect on eye health [42]. Stress may potentially raise IOP, which is a key factor in glaucoma development or progression. If left untreated, it can lead to vision loss and even blindness. Many types of glaucoma, particularly OAG, develop slowly and painlessly, with no obvious symptoms in the early stages [11]. Over time, it can cause peripheral vision loss that could be tested on VF. On the other hand, ACG can have sudden, severe symptoms like eye pain, nausea and vision impairment.

Psychoneurotic disorders are a group of psychological conditions that involve anxiety, depression, stress and emotional disturbances that can affect a person’s mental and emotional well-being. These conditions are often linked to stress and conflict and are less severe than psychotic disorders. The diagnosis of a chronic condition like glaucoma can lead to emotional and psychological challenges [43,44]. The fear of losing vision can lead to anxiety, depression or even panic attacks, which may be classified as psychoneurotic disorders [43]. People with glaucoma may develop a heightened sense of worry about their future quality of life, leading to generalized anxiety or depression which has been shown by numerous studies [44,45,46].

Both glaucoma and psychoneurotic disorders do sometimes require intensive management, often involving a combination of medical treatments and lifestyle adjustments. In glaucoma it is necessary to achieve target IOP, whether with the ATG eye drops or glaucoma surgery. In the case of psychoneurotic disorders, psychotherapy (e.g., cognitive–behavioural therapy) along with the proper medications (e.g., antidepressants or anxiolytics) could be useful to alleviate symptoms. Numerous population-based studies have shown that there is a vital connection between glaucoma and psychiatric disorders, depression and anxiety being the most common [47].

The fundamental mechanisms underlying the connection between glaucoma and psychoneurotic disorders are still unclear, i.e., complicated. However, based on the studies conducted so far, several clear directions can be found as to how this happened. Usually, patients react to a decrease in visual acuity and a decrease, i.e., a loss in the width of the visual field. This gradually leads to the inability to carry out everyday activities, and it is also impossible for some patients to continue their previous occupation. Because of the above, the possibility of earning money is reduced, and all this leads to an increase or worsening of already existing anxiety and depressive disorders. However, the worst consequence that everyone fears is still permanent blindness. Likewise, some psychiatric medications can have an effect on the existing eye condition, but on the other hand, medications used in the treatment of glaucoma can have neuropsychiatric effects [44].

Glaucoma and depression can be closely linked, with the challenges of living with glaucoma contributing to feelings of sadness, hopelessness and despair. Depression is common among individuals with chronic health conditions, and glaucoma is no exception. Previous studies have tested the level of depression using different depression scales. In their systematic review and meta-analysis, Groff et al. concluded that the presence of depression in glaucoma patients has been determined through various studies using different questionnaires [43]. However, based on an insight into the previously published and available studies, they did not pay additional attention to the severity of anxiety and depression depending on the level of progression of glaucomatous damage, nor on the achieved target IOP, which we have clearly highlighted in our study.

Moreover, Yin et al. conducted a meta-analysis comparing numerous cross-sectional studies on the prevalence and severity of depression and anxiety in glaucoma patients. This information is very valuable for ophthalmologists as well as psychiatrists. Their results showed that glaucoma patients are easily depressed and tend to have more pronounced symptoms of depression as well as anxiety symptoms. This is fully consistent with our survey. Therefore, ophthalmologists and psychiatrists should pay more attention to the increased emotional problems of patients and try to help patients stay focused on their treatment and try to improve their quality of life [46].

A similar study was conducted by Dayal et al., where they noticed the prevalence of depression from 13% to 30% and anxiety prevalence from 6% to 25%, which supports our study. They have also mentioned that there is a strong correlation between the severity of vision loss in patients with glaucoma and the appearance of symptoms of anxiety and depression [48].

Groff et al. also attempted to determine the prevalence and severity of anxiety with a variety of questionnaires, as we did. The figures showed that about 25% of glaucoma patients have anxiety, but they also have more pronounced anxiety-related symptoms. Statistically significant levels of anxiety were noted in POAG and in ACG. Nevertheless, when they tried to determine the depression prevalence, it was about 19%, and glaucoma patients had more pronounced depression-related symptoms. In this case, the depression was more pronounced in ACG glaucoma than in other glaucoma types [43].

In a study conducted by Onwubiko et al., the authors came to similar conclusions as in our study. Looking at the participants, their study also had a predominantly female population, and most participants had advanced glaucoma, of which 44% had signs of anxiety, and approximately 76% had signs of depression [49]. Due to the advanced nature of the disease, accompanied by fear of losing visual acuity, it was significant to associate it with the presence of anxiety and depression, as well as IOP values < 21 mmHg.

In our study, we have also observed the intensity of anxiety present. Our study has shown, according to the GAD-7 that, out of 200 participants, 55 (27.5%) respondents do have a mild level of anxiety, and moderately severe or severe anxiety is recorded as present in 81 (40.5%) respondents. According to the PHQ-9 questionnaire that has been used in our survey, it can be seen that 69 (34.5%) respondents have a minimal level of depression, 38 (19%) have a moderate level and, sadly, moderately severe or severe depression was registered in 31 (15.5%) respondents. Accordingly, it is evident that the psychological burden of glaucoma patients is more pronounced in those patients with more pronounced glaucoma symptoms, i.e., in those patients who have a more advanced stage of glaucoma, although we did not find statistically significant difference in anxiety and depression presence depending on the glaucoma stage.

The prevalence of anxiety and depression among glaucoma patients is generally high. In the case of patients with advanced glaucoma, the most important factor that can lead to the development of anxiety and/or depression is the fear of a decrease and possible loss of visual acuity [49]. If better attention were paid to the psychological burdens faced by glaucoma patients, especially those with the most severe stages of glaucoma, this would ensure better patient adherence to prescribed therapy [43] and greater confidence in the course of the disease, as well as a better understanding of the disease itself.

While glaucoma is primarily an eye condition, the psychological impact that it has on individuals can lead to significant anxiety. The fear of vision loss, uncertainty about the future and the stress of managing a chronic disease can all contribute to heightened anxiety as well as depression. Managing both the physical aspects of glaucoma and the emotional toll it takes is important for overall well-being. Long-term glaucoma can also lead to the presence of depression. Healthcare providers should be aware of the mental health challenges that individuals with chronic conditions like glaucoma face and should screen for anxiety and depression regularly [48].

This study has some limitations that may impact the validity of the obtained results. Since it was designed as a cross-sectional study, it is not possible to establish causality, therefore only associations between the observed variables could be observed. Other limitations of this study would also be a rather small number of participants. Therefore, future research should increase the number of participants, i.e., have a larger sample. Although the gender distribution in this study reflects the actual patient flow in our institution during the observed period, future studies could benefit from a more balanced distribution or a gender-stratified analysis to increase the credibility of the findings. It would also be advisable to have more participants with more pronounced stages of glaucoma as well as to monitor the development of psychoneurotic disorders. With the more pronounced monitoring process, we could be able to prevent the progression of psychoneurotic disorders as well as to try to slow down the speed of glaucoma progression. Regular monitoring could also prevent the patients from premature discontinuation of regular taking the prescribed therapy.

## 5. Conclusions

In conclusion, our study enhances understanding of the correlation between glaucoma and psychoneurotic disorders. According to the bivariate logistic regression of the GAD-7, results have shown that, in predicting the higher anxiety level, the subjective feeling of being exposed to stress and being in the age in the range of 56–65 years increases the probability of higher levels of anxiety. According to the CCEI, female gender, an age of 66 years and over and the feeling of being under stress have a statistically significant correlation with prediction of the expression of FFA in glaucoma patients. Prediction of the severity of depression with bivariate logistic regression and multivariate logistic regression (stepwise method), according to the PHQ-9, suggests that there are only two predictors, which are age 66 years and older and a subjective feeling of being under stress. Moreover, according to the results of this study, glaucoma severity did not show significant association with anxiety or depression levels, which has important clinical implications for screening practices. Coordinating glaucoma care between ophthalmologists and mental health professionals can ensure comprehensive care and improve quality of life. A multidisciplinary approach to glaucoma is necessary to help reduce anxiety and depression, while ongoing medical management of glaucoma is essential to maintaining eye health.

## Figures and Tables

**Table 1:** Comparison of assessment tools used in the study by function, usability and clinical validation.

Instrument	Purpose/Primary Focus	Number of Items	Clinical Validation	Usability for Patients
CCEI (*Crown–Crisp Experiential Index)*	Assesses six domains of neurotic disorders: free-floating anxiety, phobic anxiety, obsessiveness, somatic symptoms, depression, and hysteria	48	Validated in multiple clinical populations; commonly used in psychoneurotic assessments [32]	Moderate—requires more time and cognitive effort
GAD-7 *(Generalized Anxiety Disorder-7)*	Screens for and measures severity of generalized anxiety disorder	7	Widely validated and recommended for clinical use [36]	High—short, simple, and user-friendly
PHQ-9 *(Patient Health Questionnaire-9)*	Screens for and evaluates severity of depressive symptoms	9	High clinical validity and reliability [37]	High—brief and easy to administer

**Table 2:** General and clinical characteristics of the subjects; GAD-7 and PHQ-9 scale scores and distribution of respondents according to level of anxiety/depression.

Sex [*n* (%)]	
Male	68 (34)
Female	132 (66)
Age groups [*n* (%)]	
Up to 55	22 (11.0)
56–65 years	92 (46.0)
66 and over	86 (43.0)
OAG stage [*n* (%)]	
Stage I	68 (34.0)
Stage II	65 (32.5)
Stage III	67 (33.5)
Earlier ophthalmological state [*n* (%)]	36 (18.0)
Subjective feeling under stress [*n* (%)]	93 (46.5)
Achieved IOP goal [*n* (%)]	150 (75)
Age (years) [Median (IQR)]	65 (60–68)
Length of treatment (months) [Median (IQR)]	96 (39–156)
Number of application of ATG therapy per day [Median (IQR)]	2 (1–2)
OAG stage [*n* (%)]	
Stage I	68 (34.0)
Stage II	65 (32.5)
Stage III	67 (33.5)
GAD-7 Total Score [Median (IQR)]	8 (4–12)
Level of anxiety [*n* (%)]	
Mild (0–4)	55 (27.5)
Moderate (5–9)	64 (32.0)
Moderately Severe (10–14)	62 (31.0)
Severe anxiety (15–21)	19 (9.5)
PHQ-9 Total Score [Median (IQR)]	7 (3–11)
Level of depression [*n* (%)]	
Minor (0–4)	69 (34.5)
Mild (5–9)	62 (31.0)
Moderate (10–14)	38 (19.0)
Moderately Severe (15–19)	28 (14.0)
Severe depression (20–27)	3 (1.5)

IQR—interquartile range.

**Table 3:** Differences in anxiety scores (GAD-7) in relation to respondent characteristics.

	Median (IQR) GAD-7	Difference	95% Confidence Interval	*p*
Gender				
Men	8 (4–12)	0	−1–2	0.99 *
Women	8 (4–12)			
Age groups				
Up to 55 years	5 (3–9)			0.06 ^†^
56–65 years	8 (5–12)	-	-	
66 and over	9 (4–13)			
OAG stage				
Stage I	7 (5–12)			0.91 ^†^
Stage II	8 (4–12)	-	-	
Stage III	8 (4–12)			
Feeling stressed				
No	5 (3–9)	5	3–6	**<0.001** *
Yes	11 (7–14)			
Achieved IOP target values				
No	7 (4–11)	1	0–3	0.13 *
Yes	8 (4–13)			

* Mann–Whitney U test; ^†^ Kruskal Wallis test.

**Table 4:** Correlation of GAD-7 scale results with age of subjects, length of treatment and degree of glaucoma.

	Spearman’s Rho Correlation Coefficient (*p* Value)		
	Age of the Respondent	Length of Treatment	Glaucoma Stage
GAD-7 scale—all respondents	0.133 (0.06)	0.124 (0.08)	0.001 (0.99)
GAD-7 scale			
IOP target values not achieved	−0.157 (0.28)	0.209 (0.15)	0.110 (0.45)
IOP target values achieved	**0.215** **(0.008)**	0.120 (0.14)	0.036 (0.66)

**Table 5:** Differences in depression scores (PHQ-9) in relation to respondent characteristics.

	Median (IQR)	Difference	95% Confidence Interval	*p*
Sex				
Male	7 (4–11)	0	−1–2	0.76 *
Female	7 (3–12)			
Age groups				
Up to 55	6 (3–7)	-	-	**0.03** ^†§^
56–65 years	6 (3–11)			
66 and over	8 (4–13)			
OAG stage				
Stage I	6 (4–11)			0.95 ^†^
Stage II	7 (3–11)	-	-	
Stage III	7 (3–12)			
Feeling stressed				
No	4 (3–7)	6	4–7	**<0.001** *
Yes	11 (7–15)			
Achieved IOP target values				
Not	6 (3–9)	1	−1–2	0.31 *
That	7 (4–12)			

* Mann–Whitney U test; ^†^ Kruskal Wallis test (post hoc Conover test). ^§^. At the *p* < 0.05 level, the differences are significant (up to 55 years) vs. (66 and older).

**Table 6:** Correlation of PHQ-9 scale results with the age of the subjects, length of treatment and degree of glaucoma.

	Spearman’s Rho Correlation Coefficient (*p* Value)		
	Age of Subjects	Length of Treatment	Degree of Glaucoma
PHQ-9 scale—all respondents	**0.182 (0.01)**	**0.199 (0.005)**	0.020 (0.78)
PHQ-9 scale			
IOP target values not achieved	−0.152 (0.29)	0.144 (0.32)	0.154 (0.29)
IOP target values achieved	**0.269 (0.001)**	**0.221 (0.006)**	0.035 (0.67)

**Table 7:** Mean and dispersion measures of individual subscales of the CCEI questionnaire.

	Median (IQR)	Range from Minimum to Maximum Value
Free floating anxiety (FFA)	5 (2–10)	0–16
Phobic anxiety (PHOA)	5 (4–7)	0–16
Obsessiveness	8 (6–10)	0–14
Somatic manifestations of anxiety	7 (4–10)	0–16
Depression	7 (4–9)	0–14
Hysteria	4 (2–5)	0–12

**Table 8:** Distribution of respondents according to the severity of each subscale of the CCEI questionnaire.

	**Number (%) of Respondents in Relation to the Severity of Each Subscale**	
	**Not Expressed**	**Expressed**
Free floating anxiety (FFA)	119 (59.5)	81 (40.5)
Phobic anxiety (PHOA)	133 (66.5)	67 (33.5)
Obsessiveness	158 (79.0)	42 (21.0)
Somatic manifestations of anxiety	133 (66.5)	67 (33.5)
Depression	102 (51.0)	98 (49.0)
Hysteria	146 (73.0)	54 (27.0)

**Table 9:** Correlation of the age of the subjects, duration of treatment and stage of glaucoma with the subscales of the CCEI questionnaire in all subjects, and in groups according to the achieved target IOP values.

	Spearman’s Correlation Coefficient Rho (*p* Value)		
	Age of the Respondent	Duration of Treatment	Glaucoma Stage
All respondents			
Free floating anxiety (FFA)	0.068 (0.34)	0.109 (0.12)	−0.006 (0.94)
Phobic anxiety (PHOA)	0.042 (0.56)	0.051 (0.48)	0.049 (0.49)
Obsessiveness	0.050 (0.48)	0.050 (0.48)	**−0.147 (0.04)**
Somatic manifestations of anxiety	**0.190 (0.01)**	**0.164 (0.02)**	0.022 (0.76)
Depression	**0.183 (0.01)**	0.082 (0.25)	0.023 (0.75)
Hysteria	−0.021 (0.77)	0.051 (0.48)	−0.134 (0.06)
Target IOP not reached			
Free floating anxiety (FFA)	**−0.344 (0.01)**	0.022 (0.88)	−0.053 (0.71)
Phobic anxiety (PHOA)	−0.062 (0.67)	0.011 (0.94)	0.154 (0.29)
Obsessiveness	−0.057 (0.69)	0.013 (0.93)	−0.104 (0.47)
Somatic manifestations of anxiety	0.034 (0.81)	0.042 (0.77)	−0.053 (0.71)
Depression	−0.040 (0.78)	0.090 (0.54)	−0.084 (0.56)
Hysteria	−0.125 (0.39)	−0.086 (0.55)	**−0.323 (0.02)**
Target IOP achieved			
Free floating anxiety (FFA)	**0.174 (0.03)**	0.143 (0.08)	0.043 (0.60)
Phobic anxiety (PHOA)	0.077 (0.35)	0.067 (0.42)	0.052 (0.53)
Obsessiveness	0.096 (0.24)	0.068 (0.41)	−0.094 (0.26)
Somatic manifestations of anxiety	**0.222 (0.01)**	**0.197 (0.02)**	0.073 (0.37)
Depression	**0.251 (<0.001)**	0.100 (0.23)	0.100 (0.23)
Hysteria	0.016 (0.84)	0.100 (0.22)	−0.028 (0.74)

**Table 10:** Prediction of the probability of severe depression according to the PHQ-9, pronounced anxiety according to the GAD-7 questionnaire, pronounced FFA according to the CCEI and expressed PHOA according to the CCEI questionnaire (multivariate logistic regression).

	β	Wald	*p*	Odds Ratio (OR)	95% Confidence Interval
**PHQ-9**					
Age (66 and over)	0.06	5.50	**0.02**	2.03	1.03–3.99
Feeling stressed	2.27	39.3	**<0.001**	9.47	4.75–19.59
Constant	−6.27	10.7	**0.001**		
**GAD-7**					
Feeling stressed	1.79	21.4	**<0.001**	6.02	2.82–12.9
Constant	0.32	2.68	**0.04**		
**FFA (CCEI)**					
Sex (Female)	1.11	7.97	**0.005**	3.03	1.40–6.52
Feeling stressed	2.04	28.9	**<0.001**	7.70	3.66–16.21
Constant	−2.03	30.6	**<0.001**		
**PHOA (CCEI)**					
Sex (Female)	1.81	21.3	**<0.001**	6.12	2.84–13.2
Age	0.08	6.05	**0.01**	1.08	1.02–1.15
Feeling stressed	1.20	9.88	**0.002**	3.33	1.57–7.05
Constant	−6.9	2.1	**0.001**		

β—regression coefficient.

**Table 11:** Depression subscale score in relation to respondent characteristics.

	Median (IQR) Depression Subscales	Difference	95% Confidence Interval	*p* *
Sex				
Male	5 (4–8)	1	0–2	**0.006**
Female	7 (5–9)			
Age groups				
Under 55 years	5 (4–6)			**0.007** ^†§^
56–65 years	6 (4–9)	-	-	
66 and over	7 (4–9)			
Stage of OAG				
Stage I	7 (4–9)			0.94 ^†^
Stage II	6 (4–9)	-	-	
Stage III	7 (4–9)			
Feeling stressed				
No	5 (3–7)	3	2–3	**<0.001**
Yes	8 (6–10)			
Achieved IOP target values				
No	6 (4–8)	1	0–2	0.15
Yes	7 (4–9)			

* Mann–Whitney U test; ^†^ Kruskal Wallis test (post hoc Conover test). ^§^. At the *p* < 0.05 level, there are significant differences (up to 55 years) vs. (all other groups).

**Table 12:** Severity of depression in relation to characteristics of the subjects.

	Number (%) of Respondents According to Severity of Depression			*p* *
	No (*n* = 102)	Yes (*n* = 98)	Total (*n* = 200)	
Sex				
Male	37 (36.3)	31 (31.6)	68 (34)	0.49
Female	65 (63.7)	67 (68.4)	132 (66)	
Age groups				
Up to 55 years	18 (17.6)	4 (4.1)	22 (11)	**0.001**
56–65 years	51 (50)	41 (41.8)	92 (46)	
66 and over	33 (32.4)	53 (54.1)	86 (43)	
Stage of OAG				
Stage I	34 (33.3)	34 (34.7)	68 (34)	0.46
Stage II	37 (36.3)	28 (28.6)	65 (32.5)	
Stage III	31 (30.4)	36 (36.7)	67 (33.5)	
Target IOP achieved	73 (71.6)	77 (78.6)	150 (75)	0.25
Feeling stressed	34 (33.3)	59 (60.2)	93 (46.5)	**<0.001**

* χ^2^ test.

## Data Availability

All data are available and can be delivered to anyone upon request.

## Abbreviations

The following abbreviations are used in this manuscript:

ACG	Angle-closure glaucoma
ATG	Antiglaucoma
CCEI	Crown-Crisp Experiential Index
dB	Decibel
FFA	Free-floating anxiety
GAD-7	General Anxiety Disorder 7
GAT	Goldmann applanation tonometry
GCL	Ganglion cell layer
IOP	Intraocular pressure
IQR	Interquartile range
MD	Mean deviation
mmHg	Millimeters of mercury
NTG	Normal tension glaucoma
OAG	Open-angle glaucoma
OR	Odds ratio
PHOA	Phobic anxiety
PHQ-9	Patient Health Questionnaire 9
POAG	Primary open-angle glaucoma
RNFL	Retinal nerve fibre layer
VF	Visual field

## References

[1] Kaliaperumal S., Janani V.S., Menon V., Sarkar S., Behera G., Kattamani S. (2022). Study of anxiety in patients with glaucoma undergoing standard automated perimetry and optical coherence tomography—A prospective comparative study. Indian J. Ophthalmol..

[2] Jayaram H., Kolko M., Friedman D.S., Gazzard G. (2023). Glaucoma: Now and beyond. Lancet..

[3] Kang J.M., Tanna A.P. (2021). Glaucoma. Med. Clin. N. Am..

[4] Bušić M., Elabjer Kuzmanović B., Bosnar D. (2014). Seminaria Ophthalmologica.

[5] Maričić Došen V. Glaukom—Rana dijagnostika i liječenje. *Medix: Spec. Med. Dvomjesečnik,*
**2008**, *14*. https://www.medix.hr/glaukom--rana-dijagnostika-i-lijecenje_eng.

[6] Hrvatska enciklopedija (2025). glaukom—Hrvatska enciklopedija [Internet]. Hrvatska enciklopedija..

[7] Asrani S.G., McGlumphy E.J., Al-Aswad L.A., Chaya C.J., Lin S., Musch D.C., Pitha I., Robin A.L., Wirostko B., Johnson T.V. (2024). The relationship between intraocular pressure and glaucoma: An evolving concept. Prog. Retin. Eye Res..

[8] Schuster A.K., Erb C., Hoffmann E.M., Dietlein T., Pfeiffer N. The Diagnosis and Treatment of Glaucoma. Deutsches Ärzteblatt international [Internet]. 2020 Mar 27. https://www.aerzteblatt.de/10.3238/arztebl.2020.0225.

[9] Lešin Gaćina D., Jandroković S., Marčinko D., Škegro I., Vidas Pauk S., Tomić M., Škegro B., Barišić Kutija M., Ivkić P.K. (2022). Anxiety and Treatment Adherence among Glaucoma Patients during COVID-19 Pandemic and Earthquakes in Croatia. Psychiatr. Danub..

[10] Zeppieri M., Gurnani B. (2025). Applanation Tonometry. StatPearls [Internet].

[11] Mahabadi N., Zeppieri M., Tripathy K. (2025). Open Angle Glaucoma. StatPearls [Internet].

[12] Šikić J. (2003). Oftalmologija.

[13] Bradamante Ž., Bradetić T., Brzović Z. (1994). Oftalmologija.

[14] Čupak K., Gabrić N., Cerovski B. (2004). Oftalmologija.

[15] Knezović I. (2015). Oftalmologija za Studij Sestrinstva.

[16] Mandić Z. (2014). Oftalmologija.

[17] Dave S.D., Zeppieri M., Meyer J.J. (2025). Chronic Closed Angle Glaucoma. StatPearls [Internet].

[18] (2021). European Glaucoma Society Terminology and Guidelines for Glaucoma, 5th Edition. Br. J. Ophthalmol..

[19] Sihota R., Angmo D., Ramaswamy D., Dada T. (2018). Simplifying “target” intraocular pressure for different stages of primary open-angle glaucoma and primary angle-closure glaucoma. Indian J. Ophthalmol..

[20] Wu N., Kong X., Sun X. (2022). Anxiety and depression in Chinese patients with glaucoma and its correlations with vision-related quality of life and visual function indices: A cross-sectional study. BMJ Open.

[21] Agorastos A., Skevas C., Matthaei M., Otte C., Klemm M., Richard G., Huber C.G. (2013). Depression, anxiety, and disturbed sleep in glaucoma. J. Neuropsychiatry Clin. Neurosci..

[22] Kang E., Wen Z., Song H., Christian K.M., Ming G.L. (2016). Adult Neurogenesis and Psychiatric Disorders. Cold Spring Harb. Perspect. Biol..

[23] Lim M.C., Shiba D.R., Clark I.J., Kim D.Y., Styles D.E., Brandt J.D., Watnik M.R., Barthelow I.J. (2007). Personality type of the glaucoma patient. J. Glaucoma..

[24] Zimet C.N., Berger A.S. (1960). Emotional factors in primary glaucoma: An evaluation of psychological test data. Psychosom. Med..

[25] Pappa C., Hyphantis T., Pappa S., Aspiotis M., Stefaniotou M., Kitsos G., Psilas K., Mavreas V. (2006). Psychiatric manifestations and personality traits associated with compliance with glaucoma treatment. J. Psychosom. Res..

[26] Scuderi G., Pompili M., Innamorati M., Pasquale N., Pontremolesi S., Erbuto D., Mazzeo F., Venturini P., Lester D., Serafini G. (2011). Affective temperaments are associated with higher hopelessness and perceived disability in patients with open-angle glaucoma. Int. J. Clin. Pract..

[27] Igarashi Y., Sato E., Ito A., Miyauchi O., Ikejiri M., Hanawa T., Tsuyama Y., Mizota A., Fujimoto N., Adachi-Usami E. (2003). Comparison of Yatabe-Guilford personality test results in retinitis pigmentosa and glaucoma patients. Jpn. J. Ophthalmol..

[28] Bubella R.M., Bubella D.M., Cillino S. (2014). Type A behavior pattern: Is it a risk factor for open-angle chronic glaucoma?. J. Glaucoma..

[29] Çakmak H., Altinyazar V., Yilmaz S.G., Ömürlü İ.K., Kocatürk T., Yazici A., Değirmenci C., Dündar S.O., Ates H. (2015). The temperament and character personality profile of the glaucoma patient. BMC Ophthalmol..

[30] Crown S., Crisp A.H. (1994). Crown-Crispov Indeks Iskustva.

[31] Pačić Turk L., Tomašić B., Divčić B. (2013). Povezanost demografskih, socijalnih i zdravstvenih čimbenika, neuroticizma i obilježja emocionalnosti s poremećajima hranjenja. Klin. Psihol..

[32] Birtchnell J., Evans C., Kennard J. (1988). The total score of the Crown-Crisp Experiential Index: A useful and valid measure of psychoneurotic pathology. Br. J. Med. Psychol..

[33] Živkov A. (2022). Procjena Anksioznosti i Depresivnosti Studenata Fakulteta za Dentalnu Medicinu i Zdravstvo Osijek. Diploma Thesis.

[34] Islam S., Akter R., Sikder T., Griffiths M.D. (2022). Prevalence and Factors Associated with Depression and Anxiety Among First-Year University Students in Bangladesh: A Cross-Sectional Study. Int. J. Ment. Health Addict..

[35] Kroenke K., Spitzer R.L., Williams J.B.W., Löwe B. (2010). The Patient Health Questionnaire Somatic, Anxiety, and Depressive Symptom Scales: A systematic review. Gen. Hosp. Psychiatry.

[36] Spitzer R.L., Kroenke K., Williams J.B.W., Löwe B. (2006). A brief measure for assessing generalized anxiety disorder: The GAD-7. Arch. Intern. Med..

[37] Kroenke K., Spitzer R.L., Williams J.B. (2001). The PHQ-9: Validity of a brief depression severity measure. J. Gen. Intern. Med..

[38] Daniel W.W. (2012). Biostatistics: A Foundation for Analysis in the Health Sciences.

[39] Armitage P., Perry G. (2001). Statistical Methods in Medical Research.

[40] MedCalc Software Ltd., Ostend, Belgium. https://www.medcalc.org.

[41] (2019). EQUATOR The EQUATOR Network | Enhancing the QUAlity and Transparency Of Health Research [Internet]. Equator-network.org. https://www.equator-network.org/.

[42] Sabel B.A., Wang J., Cárdenas-Morales L., Faiq M., Heim C. (2018). Mental stress as consequence and cause of vision loss: The dawn of psychosomatic ophthalmology for preventive and personalized medicine. EPMA J..

[43] Groff M.L., Choi B., Lin T., Mcllraith I., Hutnik C., Malvankar-Mehta M.S. (2023). Anxiety, depression, and sleep-related outcomes of glaucoma patients: Systematic review and meta-analysis. Can. J. Ophthalmol..

[44] Zhang N., Wang J., Li Y., Jiang B. (2021). Prevalence of primary open angle glaucoma in the last 20 years: A meta-analysis and systematic review. Sci. Rep..

[45] Shin D.Y., Jung K.I., Park H.Y.L., Park C.K. (2021). The effect of anxiety and depression on progression of glaucoma. Sci. Rep..

[46] Yin J., Li H., Guo N. (2024). Prevalence of Depression and Anxiety Disorders in Patients with Glaucoma: A Systematic Review and Meta-Analysis Based on Cross-Sectional Surveys. Actas Esp. Psiquiatr..

[47] InformedHealth.org [Internet] (2006). Glaucoma: Learn More—Treatment Options for Glaucoma.

[48] Dayal A., Sodimalla K.V.K., Chelerkar V., Deshpande M. (2022). Prevalence of Anxiety and Depression in Patients With Primary Glaucoma in Western India. J. Glaucoma.

[49] Onwubiko S.N., Nwachukwu N.Z., Muomah R.C., Okoloagu N.M., Ngwegu O.M., Nwachukwu D.C. (2020). Factors associated with depression and anxiety among glaucoma patients in a tertiary hospital South-East Nigeria. Niger. J. Clin. Pract..

